# *Brucella canis* as an Underrecognized Zoonotic Threat: Human Infection, Diagnostic Challenges, and Public Health Implications

**DOI:** 10.3390/pathogens15090947

**Published:** 2026-09-07

**Authors:** Mihaela Niculae, Petru Cosmin Peştean, Victor Mazanov, George Cosmin Nadăş, Smaranda Crăciun, Stephanie Lea Oren, Ioli Kontrafouri, Haykanush Arakelyan

**Affiliations:** 1 Department of Infectious Diseases, Faculty of Veterinary Medicine, University of Agricultural Sciences and Veterinary Medicine, 400372 Cluj-Napoca, Romania; mihaela.niculae@usamvcluj.ro; 2 Department of Anesthesiology and Surgery, Faculty of Veterinary Medicine, University of Agricultural Sciences and Veterinary Medicine, 400372 Cluj-Napoca, Romania; cosmin.pestean@usamvcluj.ro; 3 Faculty of Veterinary Medicine, University of Agricultural Sciences and Veterinary Medicine, 400372 Cluj-Napoca, Romania; victor86mazanov@gmail.com (V.M.); theologia-ioli.kontrafouri@student.usamvcluj.ro (I.K.); 4 Department of Microbiology, Immunology and Epidemiology, Faculty of Veterinary Medicine, University of Agricultural Sciences and Veterinary Medicine, 400372 Cluj-Napoca, Romania; smaranda.craciun@usamvcluj.ro; 5 Department of Veterinary Pathology, Faculty of Veterinary Medicine, University of Agricultural Sciences and Veterinary Medicine, 400372 Cluj-Napoca, Romania; stephanie-lea.oren@student.usamvcluj.ro; 6 MDBiosciences Innovalora Ltd., Rehovot 7608810, Israel; 7 Department of Food Safety and Hygiene, Faculty of Veterinary Medicine and Animal Husbandry, Armenian National Agrarian University, Yerevan 0025, Armenia; arakelyan@cens.am; 8 Center for Ecological-Noosphere Studies NAS RA, Yerevan 0025, Armenia

**Keywords:** *Brucella canis*, canine brucellosis, human brucellosis, zoonosis, One Health, diagnosis, surveillance, occupational exposure, zoonotic infection

## Abstract

*Brucella canis* is a recognized zoonotic pathogen associated with human infection in a variety of epidemiological settings; however, the magnitude of its impact on human health remains uncertain because of its nonspecific clinical presentation, limited availability of species-specific diagnostic methods, and insufficient awareness among physicians, veterinarians, microbiologists, and public health professionals. Unlike classical human brucellosis caused by smooth *Brucella* species, *B. canis* infection may be overlooked, potentially leading to delayed diagnosis and incomplete case detection. This narrative review summarizes current evidence on the epidemiology, transmission pathways, occupational and non-occupational risk factors, clinical manifestations, diagnostic approaches, treatment, and public health implications of human *B. canis* infection. Particular emphasis is placed on published human cases, laboratory-acquired infections, household clusters, cases reported in different age and immune-status groups, and the biological and epidemiological characteristics of canine infection that facilitate zoonotic transmission. The review further examines the limitations of currently available microbiological, serological, and molecular diagnostic methods, the lack of harmonized surveillance systems, and the consequences of inadequate recognition from a One Health perspective. Collectively, the available evidence confirms the zoonotic potential of *B. canis* but remains insufficient to define the true burden of human infection. Improving clinical awareness, strengthening collaboration between veterinary and human health sectors, expanding access to standardized diagnostic tools, and implementing integrated One Health surveillance may improve recognition and reduce zoonotic risk.

## 1. Introduction

*Brucella canis* (*B. canis*) is recognized as one of the principal bacterial causes of reproductive disease in dogs and has gained increasing attention because of its zoonotic potential and public health relevance [1,2,3]. It is a facultative intracellular, non-motile, non-encapsulated, non-spore-forming, Gram-negative coccobacillus [4,5] that circulates primarily within domestic dog populations, which represent its main reservoir [4,6]. In canine hosts, infection is classically associated with reproductive disorders, including infertility, abortion, epididymitis, orchitis, and prostatitis [6]. These manifestations closely resemble the reproductive syndrome caused by the smooth *Brucella* species (*B. abortus*, *B. melitensis*, and *B. suis*) in livestock. However, the clinical spectrum extends beyond reproductive disease and may include prolonged bacteremia, splenitis, nephritis, discospondylitis, endocarditis, uveitis, and chorioretinitis [7,8]. Importantly, a substantial proportion of infected dogs remain clinically asymptomatic while continuing to harbor and intermittently shed the organism over extended periods [4,9]. This combination of chronic infection, asymptomatic carriage, and prolonged bacterial shedding may facilitate persistence and dissemination of *B. canis* within breeding kennels, shelters, rescue organizations, multi-dog households, and other settings where dogs are maintained in close contact, thereby increasing opportunities for transmission among animals and potential zoonotic exposure [10,11]. Evidence from studies of stray dog populations also indicates circulation of *Brucella* spp., including *B. canis*, outside owned and breeding dog populations [12].

Human infection occurs primarily through direct contact with infected dogs or exposure to contaminated biological materials, particularly reproductive materials associated with abortion [13]. *B. canis* may also be shed in reproductive secretions and urine, which can represent potential sources of exposure [14]. Laboratory-acquired infection with *B. canis* was documented in early reports, emphasizing the occupational risk associated with handling cultures and clinical specimens [15]. Human *B. canis* infection has been documented following direct or occupational exposure to infected dogs and contaminated biological materials [16,17,18,19]. In humans, infection most commonly presents as a nonspecific febrile illness characterized by fever, chills, malaise, peripheral lymphadenopathy, and splenomegaly, although severe complications, including infective endocarditis and central nervous system involvement, have also been reported [19]. Although documented human cases remain relatively uncommon, infections have been reported in both Europe and the Americas, predominantly among individuals with close household or occupational contact with infected dogs [3,16,17,18,19]. Recent One Health and veterinary investigations from Brazil and Italy further illustrate the geographical heterogeneity of canine *B. canis* circulation, exposure settings, and surveillance findings [20,21].

Owing to its nonspecific clinical presentation and the limited availability of diagnostic methods specifically validated for human *B. canis* infection, the true frequency of human infection remains uncertain. Evidence from published case reports, occupational and laboratory exposure investigations, outbreak reports, and limited serological studies indicates that the magnitude of potential underdiagnosis is difficult to quantify [1,13,19]. Recognition is further complicated by limited surveillance, low clinical suspicion, and diagnostic constraints. Despite growing recognition of its zoonotic potential, canine brucellosis is subject to heterogeneous surveillance and reporting practices across countries, limiting direct comparisons of its occurrence and public health significance [21,22]. Consequently, the available evidence confirms the zoonotic potential of *B. canis* but remains insufficient to determine the true burden of human infection [3,23].

Several recent reviews have comprehensively addressed canine brucellosis, the microbiology and epidemiology of *B. canis*, and its increasing importance in veterinary medicine and within the One Health framework [3,24,25]. However, the available evidence on human *B. canis* infection remains fragmented across individual case reports, occupational exposure investigations, laboratory-acquired infections, outbreak reports, and public health communications. The epidemiological, clinical, diagnostic, and surveillance-related evidence concerning human infection has therefore rarely been considered together within an integrated One Health framework [3].

The present narrative review integrates evidence from the veterinary, medical, and public health literature to provide a comprehensive assessment of the zoonotic implications of *B. canis* infection. By bringing together published human cases, documented exposure events, diagnostic challenges in both dogs and humans, and the veterinary characteristics that facilitate zoonotic transmission, this review critically examines the available evidence on human *B. canis* infection and the factors that complicate its recognition. Particular emphasis is placed on the interplay between clinical awareness, diagnostic limitations, and surveillance gaps within a One Health framework [26]. By synthesizing evidence across these complementary disciplines, the review identifies priorities for improving diagnosis, biosafety, reporting, surveillance, and interdisciplinary collaboration, thereby supporting evidence-based strategies to reduce human exposure and strengthen prevention at the animal–human interface.

## 2. Literature Search Strategy

A literature search was conducted to support this narrative review and to identify publications addressing the zoonotic and public health aspects of *Brucella canis* infection. Electronic searches were performed in PubMed/MEDLINE, Scopus, Web of Science, and Google Scholar using combinations of the following keywords: “*Brucella canis*”, “human infection”, “zoonosis”, “laboratory-acquired infection”, “occupational exposure”, “case report”, “family cluster”, “children”, “immunocompromised”, “diagnosis”, “surveillance”, and “One Health”. The literature was last updated on 1 September 2026.

Original research articles, case reports, outbreak investigations, surveillance reports, public health communications, and relevant review articles were considered according to their relevance to the scope of this narrative review. Veterinary studies were included when their findings directly informed zoonotic transmission, human exposure, diagnostic challenges, or prevention at the animal–human interface. Reference lists of relevant publications were also examined to identify additional pertinent studies.

A narrative approach was selected as this review objective was to integrate a heterogenous body of evidence spanning human infection, veterinary epidemiology, diagnostic methods, molecular investigations, surveillance, and regulatory frameworks within a One Health perspective, rather than to address a single predefined outcome or quantitatively synthesize a uniform group of studies. This approach also enabled incorporation of evidence that has emerged since recent reviews, including newly reported human cases and molecular investigations from 2024 to 2026, a detailed evaluation of the performance and validation limitations of diagnostic assays and reagents, as well as recent developments in surveillance and regulatory approaches, including the introduction of mandatory pre-import *B. canis* testing for certain commercially imported dogs in Great Britain in 2025. The review additionally considers *B. canis* within the current European Union regulatory framework and the implications of its absence from harmonized disease-specific surveillance and control measures. In this respect, the present review complements the 2025 scoping review of human *B. canis* infection [3] by providing an updated and broader cross-disciplinary synthesis of the available evidence.

The literature was selected on the basis of relevance to the stated objective of this narrative review and did not follow a systematic study-selection protocol; consequently, no formal risk-of-bias assessment, evidence-quality scoring or meta-analysis was performed. This approach may be subject to selection and publication bias and limits quantitative comparison across studies. The findings should therefore be interpreted as a critical synthesis of a heterogeneous evidence base rather than as quantitative estimates of incidence, prevalence, comparative risk, diagnostic performance, or therapeutic efficacy.

## 3. Veterinary Characteristics of *Brucella canis* Infection Relevant to Zoonotic Transmission

### 3.1. Clinical Complexity of Canine Brucellosis

Although canine brucellosis is classically regarded as a reproductive disease, its clinical spectrum extends beyond the reproductive tract. Similar to brucellosis caused by the smooth *Brucella* species (*B. abortus*, *B. melitensis*, and *B. suis*) in livestock, *B. canis* infection is commonly associated with abortion, embryonic death, infertility, epididymitis, orchitis, and prostatitis [2,22,27,28,29]. Extra-reproductive manifestations, including discospondylitis, polyarthritis, uveitis, lymphadenopathy, and endocarditis, have also been reported and may occur in the absence of reproductive abnormalities [2,22,27,28,29,30,31,32].

From a zoonotic perspective, this clinical variability is relevant because infected dogs without characteristic reproductive signs may not immediately raise suspicion of canine brucellosis. *B. canis* infection should therefore be considered in dogs with compatible clinical findings when supported by epidemiological risk factors, such as breeding activity, kennel residence, or recent importation [2,22,29]. Recognition of these presentations is important for initiating appropriate diagnostic testing and reducing opportunities for exposure of other dogs and humans.

### 3.2. Biological and Epidemiological Characteristics Facilitating Silent Transmission

Several biological and epidemiological features distinguish *B. canis* from many other bacterial pathogens affecting dogs and contribute to its long-term persistence within canine populations. Following infection, prolonged bacteremia may persist for several months and, in some animals, recur intermittently thereafter, providing repeated opportunities for bacterial dissemination [22,27,28].

Equally important is the fact that infected dogs may remain clinically asymptomatic. These animals may remain undetected for prolonged periods while intermittently shedding *B. canis* through reproductive secretions, semen, urine, and blood [4,14,27,28]. In contrast, abortion and associated infected materials, including aborted fetuses, placental tissues, and vaginal discharges, represent clinically apparent reproductive events that may result in substantial environmental contamination and exposure rather than silent transmission [33]. Consequently, apparently healthy dogs represent an important source of infection within breeding kennels, commercial breeding facilities, shelters, rescue organizations, and multi-dog households, where they contribute to the silent maintenance and spread of the pathogen [1,27,28,34,35]. At the population level, transmission risk is strongly influenced by epidemiological context, including the prevalence of infection in the source population, breeding practices, dog density, testing policies, and the introduction of infected animals into previously unaffected populations [34,35,36,37]. In addition, the growing movement of dogs through breeding programs, rescue organizations, shelters, and international adoption networks facilitates the geographic dissemination of the pathogen, including its introduction into new regions [1,36,37].

Recent molecular epidemiological investigations have provided additional insight into the genetic diversity and geographic relationships of *B. canis* isolates. Importantly, genomic findings reported from China originate from different host and epidemiological sources and should not be interpreted as evidence of a human transmission chain. Whole-genome analysis of the first human *B. canis* isolate reported from Yunnan Province demonstrated close phylogenetic relatedness to previously characterized strains from Zhejiang and Beijing [38]. In a separate veterinary investigation, a *B. canis* isolate recovered from an aborted canine fetus in Inner Mongolia clustered closely with strains from Zhejiang and Jiangsu, as well as with isolates from Japan and South Korea [39]. Earlier molecular epidemiological investigations of *B. canis* strains from China further demonstrated genetic diversity among isolates and provided evidence of geographic relationships among circulating strains [40].

Molecular investigations in other geographic settings have similarly demonstrated the value of genomic and molecular typing for characterizing canine *B. canis* populations and tracing their epidemiological relationships. Genomic characterization of isolates from kennel, household, and stray dogs in Chile provided insight into the diversity and population structure of circulating strains [41], while investigation of a kennel outbreak in Sweden demonstrated the utility of genetic markers for source tracing [42]. Molecular epidemiological analysis in Costa Rica revealed widespread canine *B. canis* infection together with evidence of the introduction of foreign strains [43]. In addition, genomic investigation of a canine kennel outbreak demonstrated that genetic variation may arise through within-host evolution during persistent infection [44].

Taken together, these studies demonstrate the value of WGS and complementary molecular typing approaches for investigating the genetic diversity, geographic relationships, and epidemiological origins of *B. canis.* However, phylogenetic relatedness between isolates does not by itself establish direct transmission between individual hosts or demonstrate a human transmission chain. Host species, epidemiological source, geographic origin, and available exposure information should therefore be considered together when interpreting molecular relationships among *B. canis* isolates [38,39,40,41,42,43,44].

### 3.3. Diagnostic Challenges in Dogs and Their Public Health Implications

Accurate diagnosis of canine brucellosis remains challenging because no single diagnostic test provides optimal sensitivity and specificity throughout the course of infection. The selection and interpretation of diagnostic tests should therefore take into account the stage of infection, the dynamics of bacteremia and bacterial shedding, as well as the host immune response. Serological methods, including the Rapid Slide Agglutination Test (RSAT), 2-mercaptoethanol RSAT (2ME-RSAT), agar gel immunodiffusion (AGID), and enzyme-linked immunosorbent assay (ELISA), are widely used for screening and diagnostic confirmation [45]. However, their performance varies according to the stage of infection, and they may be affected by false-positive reactions, making confirmatory testing essential for an accurate diagnosis [22,27,28,45].

Direct diagnostic methods, including blood culture, semen culture, vaginal secretion culture, and polymerase chain reaction (PCR), provide microbiological confirmation of *B. canis* infection but are also associated with important limitations [27,28,45,46,47,48,49]. Culture sensitivity may be affected by intermittent bacteremia and bacterial shedding, while isolation of viable *Brucella* requires appropriate biosafety precautions because of the occupational risk associated with laboratory handling [27,28,49]. PCR enables rapid and specific detection of *B. canis* DNA in different clinical specimens [45,46,47]; however, differences in sample type, assay design, and laboratory protocols limit direct comparison and broader standardization of molecular testing [45,46,47,48,49].

These diagnostic limitations have important implications from a One Health perspective. Delayed or missed diagnosis in dogs may allow infected animals to remain undetected within breeding kennels, shelters, rescue organizations, and households, increasing opportunities for transmission to other dogs and potential human exposure. Appropriate selection and interpretation of veterinary diagnostic tests are therefore important not only for disease control in dogs but also for reducing exposure at the animal–human interface [1,26,49].

## 4. Published Human Cases: An Integrated Epidemiological Analysis

### 4.1. Characteristics of Documented Human Brucella canis Infection

Compared with human brucellosis caused by *Brucella melitensis*, *B. abortus*, or *B. suis*, documented human infections with *B. canis* remain relatively uncommon. However, the number of published cases cannot be considered a reliable estimate of incidence because available evidence is derived largely from case reports, outbreak investigations, and limited exposure studies [3,50,51,52,53,54]. Accordingly, documented infection should be distinguished from serological evidence of exposure and from suspected but unconfirmed underdiagnosis.

Published evidence identifies occupational contact with dogs and canine biological materials as an important setting for human exposure [50,51,52]. Individuals at increased risk include veterinarians, veterinary nurses and technicians, kennel personnel, dog breeders, animal shelter employees, laboratory personnel, and other professionals who have frequent contact with dogs or canine biological specimens as part of their occupational activities [1,17,50,51,52,55].

Human infection may occur through direct contact with infected dogs or exposure to contaminated biological materials, particularly blood and reproductive materials, including vaginal discharges, semen, aborted fetuses, and placental tissues [51,52,54]. These exposure pathways reflect important characteristics of canine infection, including prolonged bacteremia and bacterial shedding [4,14,27,28].

Human infection is not restricted to occupational settings. Documented cases and outbreak investigations also demonstrate transmission associated with close contact with infected companion dogs [19,51,54]. Thus, the available evidence supports considering the nature and intensity of exposure to infected dogs and their biological materials rather than occupational status alone when assessing zoonotic risk. Exposure events have also been documented in unusual public settings, including contact with an infected pregnant dog during international air travel [56].

### 4.2. Laboratory-Associated Infections: Occupational Risk and Biosafety Implications

Brucellosis is widely recognized as one of the most frequently reported laboratory-acquired bacterial infections worldwide [57,58]. Although most laboratory-associated infections are caused by the smooth *Brucella* species (*B. melitensis*, *B. abortus*, and *B. suis*), occupational exposure to *B. canis* has also been documented, demonstrating that this species likewise poses a laboratory hazard [15,16,17,18,59].

Laboratory personnel represent an occupational group at particular risk because *Brucella* spp. can establish infection following exposure to very small inoculum, particularly through infectious aerosols generated during routine microbiological procedures. Manipulation of blood cultures, centrifugation, preparation of bacterial suspensions, pipetting, subculturing, biochemical identification, and other laboratory procedures may generate aerosols before *Brucella* is recognized as the causative agent. Consequently, occupational exposure may occur during the routine processing of unsuspected clinical specimens rather than during the handling of confirmed *Brucella* isolates, when appropriate biosafety measures are already in place [59,60].

Early laboratory-acquired *B. canis* infections were described by Morisset et al. in two laboratory workers exposed while preparing *B. canis* antigen. Although the presumed route of infection was inoculation through contaminated skin abrasions, the two cases differed markedly in clinical severity, ranging from mild fatigue to systemic illness with persistent bacteremia, lymphadenopathy, and splenomegaly. Both patients recovered following tetracycline therapy. These early observations provided evidence of the zoonotic potential of *B. canis* and illustrated the variable clinical presentation of laboratory- associated infection [15].

More than three decades later, Wallach et al. reported another laboratory-acquired *B. canis* infection in a technician who manipulated viable bacteria outside a biological safety cabinet and without appropriate personal protective equipment. The patient developed a febrile systemic illness, and the diagnosis was confirmed by blood culture and species-specific serology. This case illustrated the occupational risk associated with inadequate biosafety practices during manipulation of viable *B. canis* [16].

More recent reports indicate that the greatest laboratory hazard often arises not from the manipulation of recognized *Brucella* cultures but from the delayed identification of unsuspected clinical isolates. Dentinger et al. described a large laboratory exposure following the diagnosis of pediatric *B. canis* infection in New York City. Before *Brucella* was suspected, the child’s positive blood cultures were processed according to routine laboratory procedures on an open bench, resulting in the potential exposure of 31 laboratory personnel [17]. Although no secondary laboratory-acquired infections occurred, the incident required extensive public health intervention, including risk assessment, post-exposure prophylaxis for high-risk personnel, and six months of active symptom surveillance. This investigation highlighted the substantial biosafety and public health consequences of delayed laboratory recognition of *Brucella* spp. [17].

Similarly, Ahmed-Bentley et al. reported a high-risk laboratory exposure after a *B. canis* isolate was manipulated on an open laboratory bench before the organism had been correctly identified. Seventeen clinical microbiology personnel were classified as having high-risk exposure, and one laboratory worker subsequently had a positive serological result for *B. canis*. Although no clinically apparent disease occurred, the investigation reinforced the importance of prompt identification of the organism, appropriate biosafety practices, and clinical and serological follow-up for personnel involved in high-risk laboratory incidents [18].

Collectively, these reports demonstrate that laboratory-associated *B. canis* exposure may occur through multiple routes, including direct manipulation of viable cultures, accidental inoculation, contact with contaminated materials, and generation of infectious aerosols during routine laboratory procedures. Because laboratory confirmation of brucellosis involves multiple manipulations, including culture, subculture, biochemical characterization, and species identification, minimizing occupational risk requires strict adherence to biosafety protocols, prompt recognition of suspected *Brucella* isolates, and effective communication between clinicians, veterinarians, and microbiology laboratories [60].

### 4.3. Beyond Occupational Exposure: Broader Epidemiological Settings

Beyond established occupational settings, evolving patterns of dog ownership and the increasing movement of dogs through rescue and adoption networks may expand the range of individuals potentially exposed to *B. canis* [35,36,37].

Increasing public involvement in dog rescue, fostering, shelter activities, and international adoption may result in close contact with dogs of unknown infectious status. Such contact may include exposure to potentially infectious biological materials, particularly reproductive secretions, blood, urine, aborted fetuses, and placental tissues. Because infected dogs may remain clinically asymptomatic, exposure can occur before canine brucellosis is recognized [1,3,10,19,35,36,37]. A recent report from China further illustrates this risk. Ge et al. described probable zoonotic transmission from an asymptomatic adopted dog to its owner following unprotected handling of aborted tissues. Serological, qPCR, metagenomic, and genomic investigations detected *B. canis* and *B. melitensis* genetic material in the dog, highlighting both the zoonotic implications of companion-animal exposure and the diagnostic complexity of Brucella infections [61].

An illustrative example of this epidemiological context was provided by Schiavo et al. (2024), who investigated *B. canis* infection among individuals with animal hoarding disorder and their dogs using a One Health approach [20]. Although no serological evidence of human infection was detected, serological and molecular testing confirmed the circulation of *B. canis* in several households. The study therefore documents a setting in which prolonged human contact with infected dog populations may occur, while also illustrating that evidence of canine infection or exposure opportunity should not be interpreted as evidence of human infection [20].

Collectively, these observations indicate that potential zoonotic exposure to *B. canis* is not confined to traditional occupational settings and may also occur in domestic, rescue, foster-care, shelter, and adoption contexts [7,11,20,35,36,37]. Recent surveillance data from Zhejiang Province further emphasize the broader importance of non-occupational exposure in brucellosis control, with 499 non-occupational cases reported during 2018–2024 and multiple exposure pathways identified; however, these data were not specific to *B. canis* [62].

**Figure 1:**
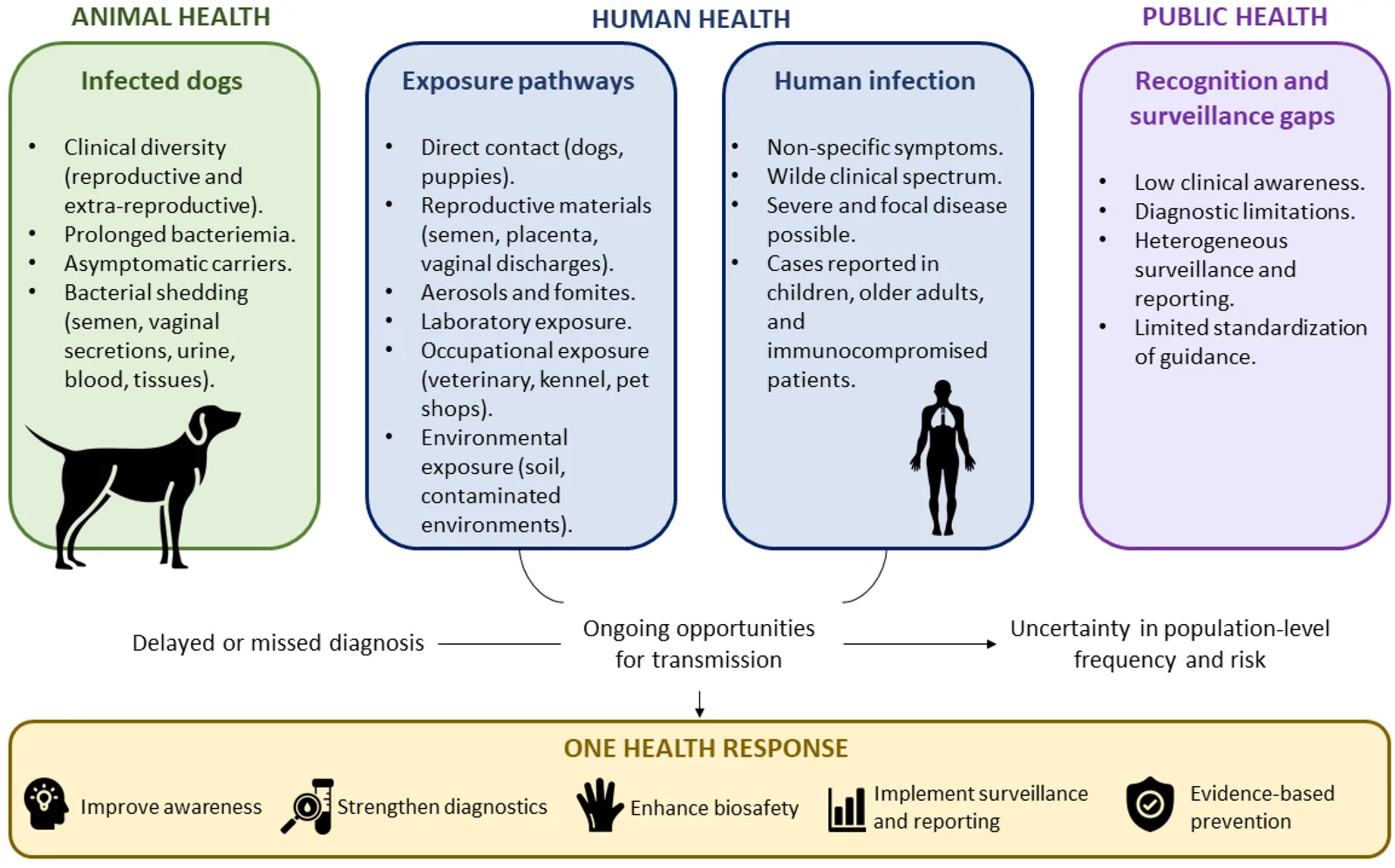
One Health framework illustrating the epidemiology of *Brucella canis* infection. The figure summarizes the links between canine infection, routes of human exposure, public health consequences, and coordinated One Health interventions aimed at improving surveillance, diagnosis, biosafety, and prevention.

These epidemiological settings illustrate the close relationship between canine infection and opportunities for human exposure and support consideration of *B. canis* within an integrated One Health framework (Figure 1). Coordinated veterinary, medical, laboratory, and public health approaches are therefore relevant to surveillance and prevention at the animal–human interface [1,20,26].

### 4.4. Household Clusters and Unusual Exposure Events

Although most documented infections involve isolated individuals, household transmission has also been reported. Lucero et al. described a family cluster involving three adults and three children, demonstrating that prolonged exposure to a single infected dog was associated with multiple human infections within the same household [54]. More recently, Williams et al. described an unusual exposure event following the abortion of an infected dog during an international commercial flight. The subsequent multidisciplinary public health investigation highlighted the challenges of managing unexpected *B. canis* exposure events in public settings and underscored the importance of coordinated responses involving veterinary, laboratory, and public health authorities [56]. A recent scoping review identified 24 studies describing clinical *B. canis* infection in 68 individuals; among cases with a known or suspected exposure source, 80% were associated with dogs and 20% with laboratory exposure [3].

Table 1 summarizes the human cases and reports identified in the literature reviewed here and should not be interpreted as a systematic or exhaustive registry of all human *B. canis* infections.

Among the 21 reports summarized in Table 1, nine originated from the United States, four from Argentina, three from Japan, and one each from Iran, Canada, the Netherlands, South Africa, and Chile [74,75]. The recent Chilean series included 10 patients diagnosed by ME-RSAT serology, with dog exposure reported in most cases; importantly, blood cultures were negative in the patients tested, illustrating the heterogeneity in the level of diagnostic evidence across published reports [75]. Most reports described direct household or occupational exposure to dogs or canine biological materials, whereas laboratory-associated exposure was documented in early reports. Cases involved children and adolescents, adults, and older individuals. Culture contributed to diagnostic confirmation in 16 of the 21 reports, whereas several reports relied on serological evidence alone or included mixed levels of diagnostic evidence. Nonspecific febrile illness and bacteremia were common clinical presentations, while severe focal manifestations, including endocarditis, osteoarticular involvement, and neurobrucellosis, were also reported.

**Figure 2:**
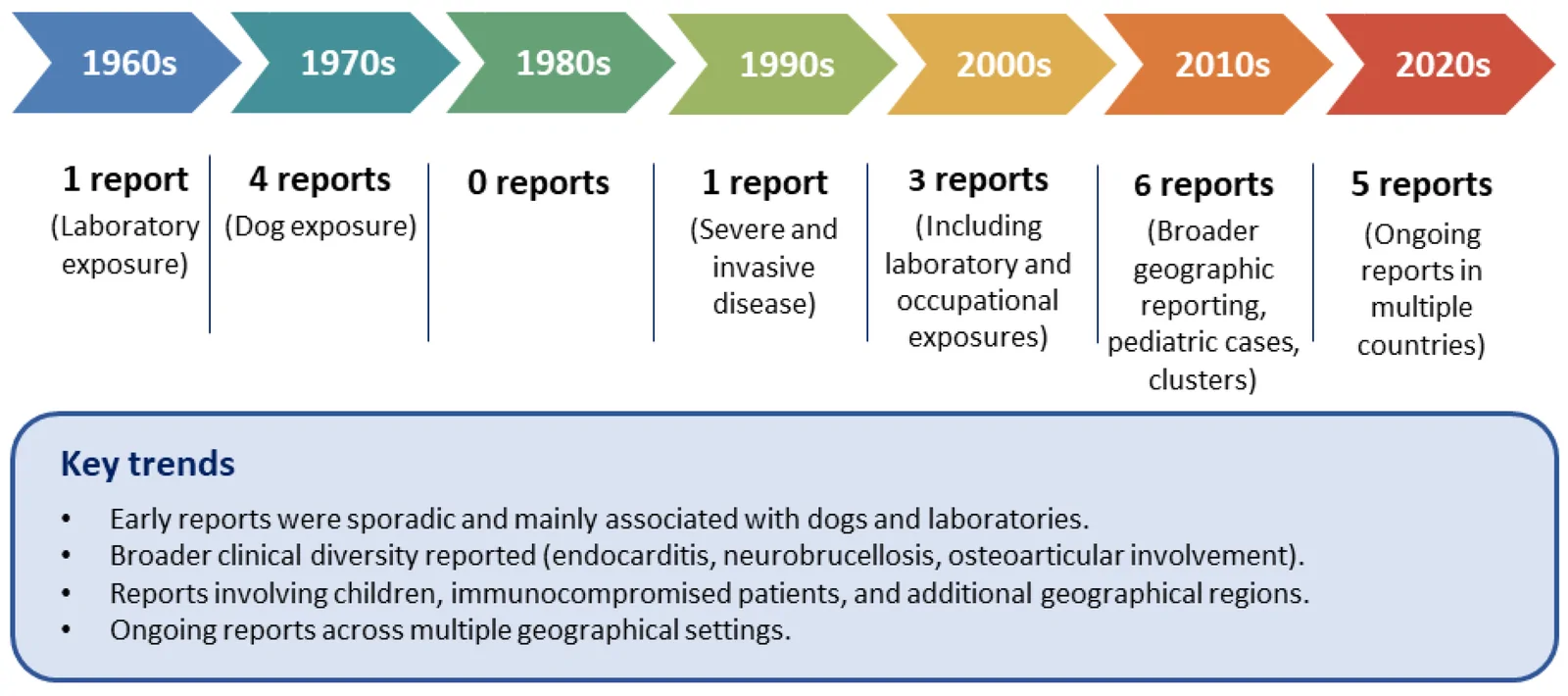
Timeline illustrating the evolution of reported human *Brucella canis* infections from the 1960s to the 2020s. The figure summarizes the temporal distribution of published reports, the broadening geographical range of reported cases, and the diversity of clinical presentations described in the literature.

Although individual case reports remain relatively uncommon, the temporal distribution of published reports shows that human *B. canis* infection has been documented over more than five decades (Figure 2). Early reports were sporadic and were primarily associated with laboratory exposure or direct contact with infected dogs. More recent reports include cases from multiple geographical regions and a broader range of epidemiological and clinical settings, including severe focal manifestations, pediatric cases, household clusters, and infections in immunocompromised individuals. However, the available literature consists predominantly of case reports, case series, and exposure investigations and therefore does not allow assessment of temporal trends in incidence. The apparent increase in the number and diversity of published reports may reflect greater clinical awareness, improved diagnostic capacity, and increased reporting rather than a true increase in human infection. Consequently, the available evidence supports the occurrence of human *B. canis* infection across diverse settings but remains insufficient to quantify its population-level burden [13,19].

## 5. Clinical Manifestations and Disease Spectrum

Human *B. canis* infection lacks a pathognomonic clinical presentation and most commonly manifests as a nonspecific febrile illness. Patients typically present with flu-like symptoms, including intermittent or undulating fever, chills, malaise, fatigue, headache, and generalized weakness, frequently accompanied by peripheral lymphadenopathy, hepatomegaly, and splenomegaly [3,19,53]. Because these manifestations overlap with those of numerous viral, bacterial, and inflammatory diseases, *B. canis* infection may not be considered in the initial differential diagnosis unless a history of contact with infected dogs or relevant occupational exposure is specifically identified. As a result, diagnosis may be delayed, postponing microbiological confirmation and the initiation of appropriate antimicrobial therapy [19,53].

An additional challenge in estimating the true burden of human *B. canis* infection is that exposure does not invariably result in clinically apparent disease. Evidence from serological surveys indicates that antibodies against *B. canis* may be detected in asymptomatic individuals or in those with only mild, nonspecific illness that does not prompt medical evaluation. This pattern has been documented among veterinarians and other individuals with frequent occupational exposure to infected dogs, in whom antibodies against *B. canis* have been detected despite the absence of compatible clinical manifestations [50].

Furthermore, estimates of human serological exposure and infection are strongly influenced by the serological methods used [50]. In the study by Krueger et al., seroprevalence among occupationally exposed individuals was 10.8% when screened using the Rapid Slide Agglutination Test (RSAT) but decreased to 3.6% after confirmation with the more specific 2-mercaptoethanol Rapid Slide Agglutination Test (2-ME RSAT or ME-RSAT) [50]. This discrepancy reflects differences in test specificity and highlights the importance of interpreting serological findings within the context of the diagnostic algorithm employed. Consequently, serological evidence of exposure and the limitations of currently available serological assays complicate the interpretation of seroprevalence as a measure of human infection [50,53]. Although many reported human *B. canis* infections have been mild or nonspecific, severe invasive disease has also been documented. Published cases include persistent bacteremia [70], infective endocarditis [67], septic arthritis [3], and central nervous system involvement, including neurobrucellosis and meningoencephalitis [73]. These reports illustrate the broad clinical spectrum of human *B. canis* infection and the potential for serious focal or systemic manifestations.

Published evidence suggests that certain population groups may warrant particular clinical attention following exposure to *B. canis*. Although the number of documented human cases remains limited, clinically significant infections have been reported in children [17,54], immunocompromised individuals, including patients with HIV infection [69,76], and older adults [13,18,73]. However, the available evidence is derived primarily from individual case reports and is insufficient to determine whether these groups are at increased risk of infection or severe disease.

Children are among the populations in which *B. canis* infection has been documented following close contact with household pets, particularly puppies [17,54]. The pediatric cases reported to date demonstrate that *B. canis* infection may initially mimic common childhood illnesses, potentially delaying diagnosis. These reports also illustrate the potential role of infected puppies acquired through commercial breeding or pet trade networks as a source of household transmission [17].

Immunocompromised individuals are another group in whom *B. canis* infection has been reported. Although only a few cases have been described, the available evidence is insufficient to determine whether impaired immunity increases susceptibility to infection or the risk of severe disease [69,76]. Lucero et al. reported *B. canis* infection in a 36-year-old man with advanced HIV infection who presented with prolonged fever, asthenia, malaise, headache, and respiratory symptoms [69]. Because the clinical presentation was initially compatible with a more common bacterial respiratory infection, empirical antimicrobial therapy was started before blood cultures identified *B. canis*. Following microbiological confirmation, targeted antimicrobial treatment resulted in complete clinical recovery [69]. This case illustrates how the nonspecific presentation of *B. canis* infection in an immunocompromised patient may mimic other infectious diseases, potentially delaying diagnosis [69].

Clinically significant *B. canis* infection has also been reported in older adults. Although relatively few cases have been reported, published evidence demonstrates clinical heterogeneity, ranging from prolonged nonspecific febrile illness to severe systemic and neurological involvement. Majzoobi et al. reported *B. canis* infection in a 68-year-old woman whose prolonged febrile illness was initially treated as urosepsis [13]. Routine serological tests for brucellosis were negative, and the diagnosis was established only after blood culture and polymerase chain reaction (PCR) confirmed *B. canis* infection. This case highlights the limitations of conventional serological testing in human *B. canis* infection [13].

More recently, Ishihara et al. reported a rare case of neurobrucellosis in a 68-year-old man presenting with meningoencephalomyelitis. The diagnosis was established by *B. canis* serology in combination with abnormal cerebrospinal fluid findings. This case illustrates that central nervous system involvement can represent a severe manifestation of human *B. canis* infection [73].

Ahmed-Bentley et al. also reported *B. canis* infection in a 70-year-old woman receiving immunomodulatory therapy for psoriatic arthritis. The diagnosis was established by blood culture after 3.5 days of incubation, and the patient responded well to targeted antimicrobial therapy. This case further illustrates the diagnostic challenges associated with human *B. canis* infection [18].

Collectively, these reports indicate that older adults may present with prolonged febrile illness and nonspecific systemic manifestations that may mimic more common infectious, inflammatory, or malignant conditions [13,18,73]. Delayed diagnosis and negative routine serological findings have been described in published cases [3,13]. Although the available evidence remains limited, *B. canis* infection should be considered in the differential diagnosis of older patients with persistent febrile illness and relevant epidemiological exposure, particularly when initial diagnostic investigations are inconclusive [3,13,18,73].

Taken together, the available evidence suggests that the clinical presentation of *B. canis* infection may be influenced by the intensity and nature of exposure and by host-related factors. While exposure to infected dogs or contaminated canine materials is a major epidemiological context for transmission, age, immune status, and underlying health conditions have been described in patients with clinically significant disease but their contribution to disease severity and complications remains uncertain. Recognition of these clinical and epidemiological contexts suggests that the clinical presentation of *B. canis* infection may be influenced by host-related factors [3,69,73].

## 6. Laboratory Diagnosis of Human *Brucella canis* Infection

Laboratory confirmation of human *B. canis* infection can be more challenging than the diagnosis of classical human brucellosis caused by the smooth *Brucella* species [3]. These challenges arise from the nonspecific clinical presentation of the disease, limited clinical awareness, the absence of standardized diagnostic algorithms, and the restricted availability of diagnostic assays specifically developed for *B. canis*. Consequently, laboratory confirmation often requires the integration of microbiological, serological, and molecular methods, each with distinct strengths and limitations [3].

Three complementary diagnostic approaches are currently available: (i) direct isolation of the organism by culture, (ii) indirect diagnosis through the detection of anti-*B. canis* antibodies, including agglutination-based methods described for human testing [77], and (iii) molecular detection using polymerase chain reaction (PCR)-based assays [78] (Table 2).

Isolation of *B. canis* from blood culture remains the reference method for confirming human infection because it provides direct microbiological evidence and enables species identification. Several published human cases have been diagnosed using blood culture [16,17]. However, the diagnostic sensitivity of blood culture is limited by the intermittent or low-grade bacteremia that frequently characterizes *B. canis* infection, particularly in prolonged or chronic infection, when circulating bacterial levels may be reduced [78]. In addition, manipulation of viable isolates poses a recognized laboratory biosafety risk, emphasizing the importance of appropriate biosafety procedures and early recognition of suspected *Brucella* isolates [59,78].

Serological testing remains the most widely used diagnostic approach for human brucellosis. However, most commercially available assays were developed using smooth *Brucella* antigens. Smooth *Brucella* species possess an immunodominant O-polysaccharide (O-chain) as part of their lipopolysaccharide, whereas the rough species *B. canis* naturally lacks this O-chain. Consequently, conventional serological assays based on smooth *Brucella* lipopolysaccharide have limited utility for detecting *B. canis* infection. Several alternative serological assays using *B. canis*-relevant antigens have been evaluated. Lucero et al. (2005) reported that an indirect ELISA achieved 100% sensitivity in 17 sera from patients with culture-confirmed *B. canis* infection or close contact with culture-positive dogs [53].

More recently, Sánchez-Jiménez et al. (2020) demonstrated that an iELISA combining the recombinant PdhB and Tuf antigens achieved a sensitivity of 98% and a specificity of 73% when compared with PCR in the human serum samples evaluated, highlighting the potential of recombinant antigen-based assays to improve the serological diagnosis of human *B. canis* infection [79]. Importantly, seropositivity should be interpreted as immunological evidence of exposure and, in the absence of compatible clinical findings or direct microbiological confirmation, should not be considered sufficient on its own to establish active *B. canis* infection [50,53]. PCR-based methods provide rapid and highly specific detection of *Brucella* DNA and may facilitate species identification, particularly when culture is negative, or antimicrobial therapy has already been initiated. Diagnostic performance depends substantially on the genomic target and assay design. Common targets used for *Brucella* detection include *bcsp31*, the insertion sequence IS711, and outer-membrane protein loci such as *omp2a/omp2b* [78]. The *bcsp31* gene is among the most widely used genus-level targets, whereas IS711-based real-time PCR may provide greater analytical sensitivity because this insertion sequence is present in multiple copies, although copy-number and sequence variability can affect assay performance [75]. Accordingly, diagnostic performance may vary according to the molecular target, assay format, and clinical specimen, and findings from human brucellosis caused predominantly by smooth *Brucella* species should not be extrapolated directly to human *B. canis* infection [78]. PCR assays specifically designed for *B. canis* have also been developed, but their validation has largely involved canine samples rather than human clinical specimens [45,46,47]. Their routine clinical use remains limited by the lack of standardized protocols, commercially available assays, and large-scale clinical validation studies [59,78].

Matrix-assisted laser desorption/ionization time-of-flight mass spectrometry (MALDI-TOF MS) can provide rapid identification of cultured *Brucella* isolates, but its performance depends strongly on the reference database used. In human *B. canis* infection, MALDI-TOF MS may identify the isolate at the genus level but may fail to reliably distinguish *B. canis* from closely related *Brucella* species, requiring confirmatory molecular or other species-level testing [52,59].

**Figure 3:**
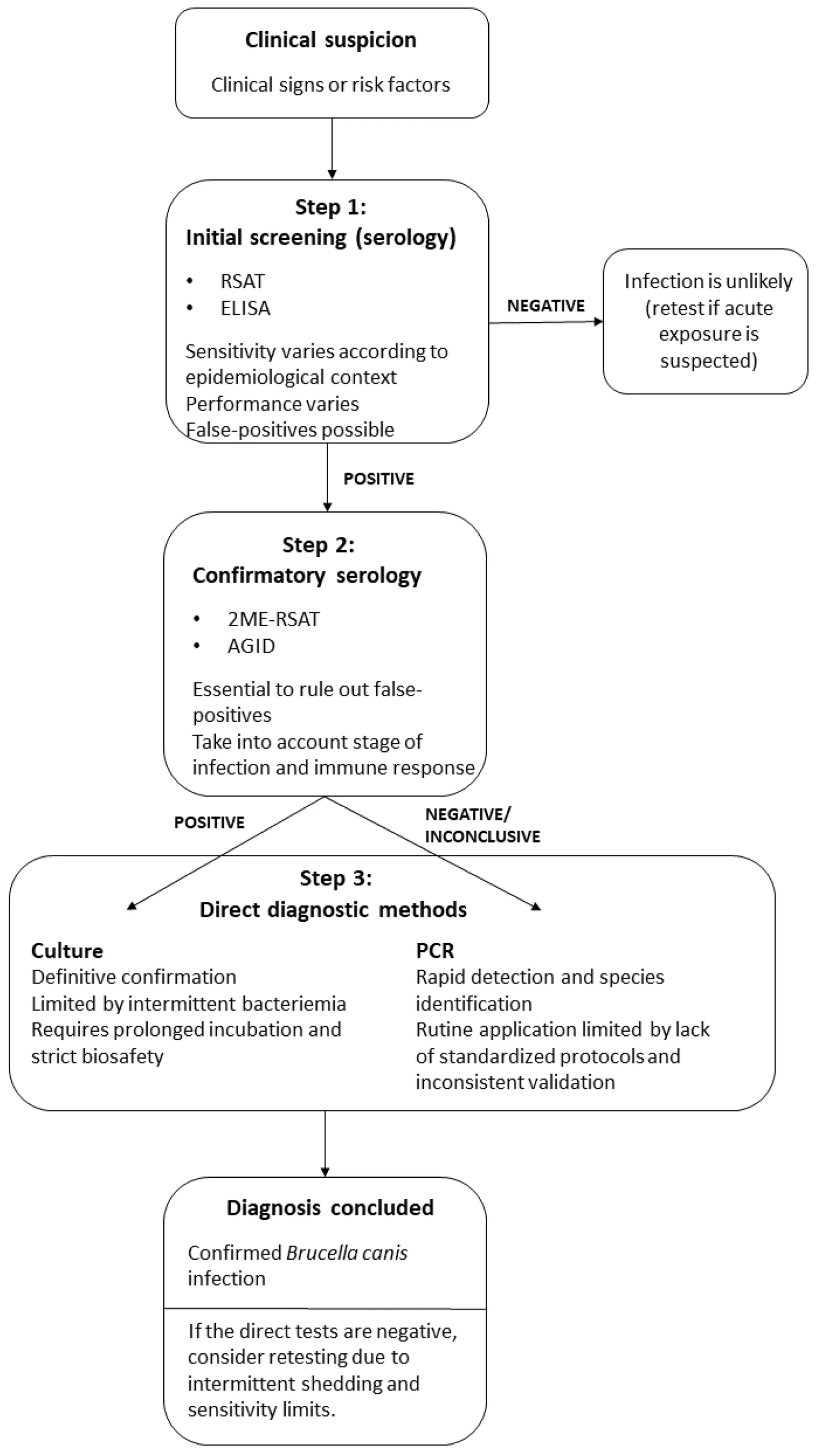
Conceptual diagnostic decision framework for *Brucella canis* derived from the diagnostic approaches discussed in the reviewed literature; the framework has not been clinically validated as a diagnostic algorithm.

Based on the reviewed literature, a proposed diagnostic decision framework for *B. canis* is summarized in Figure 3.

This framework should be interpreted as a conceptual synthesis of the available literature rather than as a clinically validated diagnostic algorithm. Serological positivity alone indicates immunological evidence of exposure and does not necessarily establish active infection, particularly in asymptomatic individuals. Conversely, a negative culture does not exclude infection because bacteremia may be intermittent or low-grade and bacterial growth may require prolonged incubation. Similarly, a negative PCR result should be interpreted in the context of specimen type, timing of sampling, and assay design, as molecular methods remain incompletely standardized for human *B. canis* infection. Diagnostic interpretation should therefore integrate clinical findings, exposure history, and complementary laboratory results [3,53,78].

Collectively, the available evidence does not identify a single laboratory method that currently provides optimal sensitivity and specificity for the diagnosis of human *Brucella canis* infection. Blood culture remains the reference standard but is limited by variable sensitivity, prolonged incubation, and biosafety concerns. Serological diagnosis requires careful interpretation because most conventional assays were developed for the smooth *Brucella* species, whereas molecular methods remain insufficiently standardized and have limited clinical validation specifically for human *B. canis* infection. Together, these diagnostic challenges may contribute to delayed recognition of human *B. canis* infection and complicate estimation of its true frequency [3,59,78]. Improving laboratory diagnosis will therefore require greater clinical awareness, the development of standardized and clinically validated *B. canis*-specific diagnostic assays, and closer collaboration between clinicians, microbiologists, veterinarians, and public health professionals within a One Health framework [26].

## 7. Treatment of Human *Brucella canis* Infection and Its Outcome

No evidence-based treatment guidelines specific for human *B. canis* infection are currently available, and therapeutic approaches are largely based on regimens used for human brucellosis caused by the smooth *Brucella* species. Thus, therapy generally relies on antimicrobials active against intracellular bacteria, namely tetracyclines such as doxycycline administered alone or, preferably, combined with rifampicin or an aminoglycoside such as streptomycin or gentamicin [3]. The antimicrobial treatment is typically continued for several weeks in connection with *Brucella*’s potential intracellular persistence leading to therapy failure or disease relapse.

The recent scoping review by Weese and Weese [3] identified considerable variation in the antimicrobial management of human *B. canis* infection. Specific treatment information was available for only 30 individuals; tetracyclines, alone or in combination, were the most frequently used antimicrobials (21/30, 70%), followed by rifampin (9/30, 30%), trimethoprim-sulfamethoxazole (7/30, 23%), and aminoglycosides (7/30, 23%). Reported combinations included doxycycline with rifampin, streptomycin, or gentamicin, among several other therapeutic approaches [3]. This diversity reflects both the absence of standardized recommendations for human *B. canis* infection and the heterogeneous clinical circumstances in which these cases have been recognized. Treatment duration also varies and cannot be generalized from the available cases. Although 6-week regimens have been successfully used in several culture-confirmed infections [70,72], substantially longer therapy may be required in complicated or focal disease. Nomura et al. [70] reported two patients with blood culture-confirmed *B. canis* infection who received doxycycline-based treatment for 6 weeks, combined with streptomycin during the initial 2 weeks in one patient and with rifampin throughout treatment in the other. In contrast, in the case of *B. canis* meningoencephalomyelitis reported by Ishihara et al. [73], doxycycline was administered for 96 days, with streptomycin during the initial 16 days. The prolonged course pointed out the severity of neurological involvement and the gradual improvement of cerebrospinal fluid and serological findings; the patient ultimately achieved clinical and cerebrospinal fluid improvement without neurological sequelae during long-term follow-up [73]. Thus, although 6-week treatment courses have been successful in several reported infections, the current evidence does not support a uniform 6–8-week duration for human *B. canis* infection, particularly in patients with focal or complicated disease.

Primary case reports further underline the variability of therapeutic approaches while generally indicating favorable clinical responses. Lucero et al. [69] described an HIV-positive patient with culture-confirmed *B. canis* infection who was successfully treated with doxycycline combined with ciprofloxacin. Treatment may require further adaptation in pregnancy, when commonly used tetracycline-based regimens are unsuitable. In the pregnancy-associated case reported by Dunn et al. [72], ceftriaxone, rifampin, and gentamicin were initially administered during the third trimester. Gentamicin was discontinued after 7 days, and rifampin was subsequently withdrawn because of elevated liver enzymes; following delivery, treatment was modified to doxycycline and ceftriaxone. The patient completed a total 6-week course calculated from the first negative blood cultures, with a favorable maternal outcome and no reported infection in the infant [72]. These cases illustrate that successful treatment has been achieved using different antimicrobial combinations, but they represent individualized clinical experiences rather than evidence of comparative efficacy between regimens.

Unlike canine brucellosis, which is characterized by persistent infection and frequent therapeutic failure despite prolonged antimicrobial treatment [27,80], human *B. canis* infection has generally shown favorable clinical outcomes [3]. In the 2025 scoping review, outcome information was available for 35 individuals, all of whom were reported to have a clinical response, and no deaths were identified [3]. Also, published case reports indicate generally favorable clinical outcomes following antimicrobial treatment in both uncomplicated infections and severe manifestations, including persistent bacteremia [70], neurobrucellosis [73], and infective endocarditis [65].

Nevertheless, these findings require cautious interpretation as the evidence consists predominantly of individual case reports and small case series, treatment regimens and durations are heterogeneous, and microbiological and long-term follow-up are inconsistently reported. Moreover, clinical recovery should not necessarily be considered equivalent to microbiological eradication. Persistent subclinical bacteremia has been documented despite clinical resolution [3]. This apparent contrast between canine and human infection should not be interpreted as evidence that *B. canis* is intrinsically more readily eradicated in humans. The number of documented human infections is small, microbiological clearance has not been systematically assessed, follow-up is inconsistent, and persistent bacteremia has also been documented in humans. Differences in case ascertainment, clinical presentation, host response, treatment practices, and post-treatment monitoring may therefore contribute to the apparently more favorable outcomes reported in humans.

Importantly, there are also no controlled studies directly comparing therapeutic response in human *B. canis* infection with brucellosis caused by smooth Brucella species, particularly *B. melitensis*, despite the fact that treatment of *B. canis* infection is largely extrapolated from experience with these organisms. Consequently, the available evidence does not establish whether *B. canis* differs from other Brucella species in microbiological clearance, treatment response, or recurrence in humans. The generally favorable outcomes reported to date are encouraging, but the absence of randomized controlled trials, standardized treatment protocols, and systematic long-term follow-up prevents definition of an optimal antimicrobial combination or duration of therapy. Further clinical evidence is therefore required to establish criteria for microbiological cure, determine the risk of persistence or recurrence, and develop species-specific recommendations for the treatment and follow-up of human *B. canis* infection.

**Figure 4:**
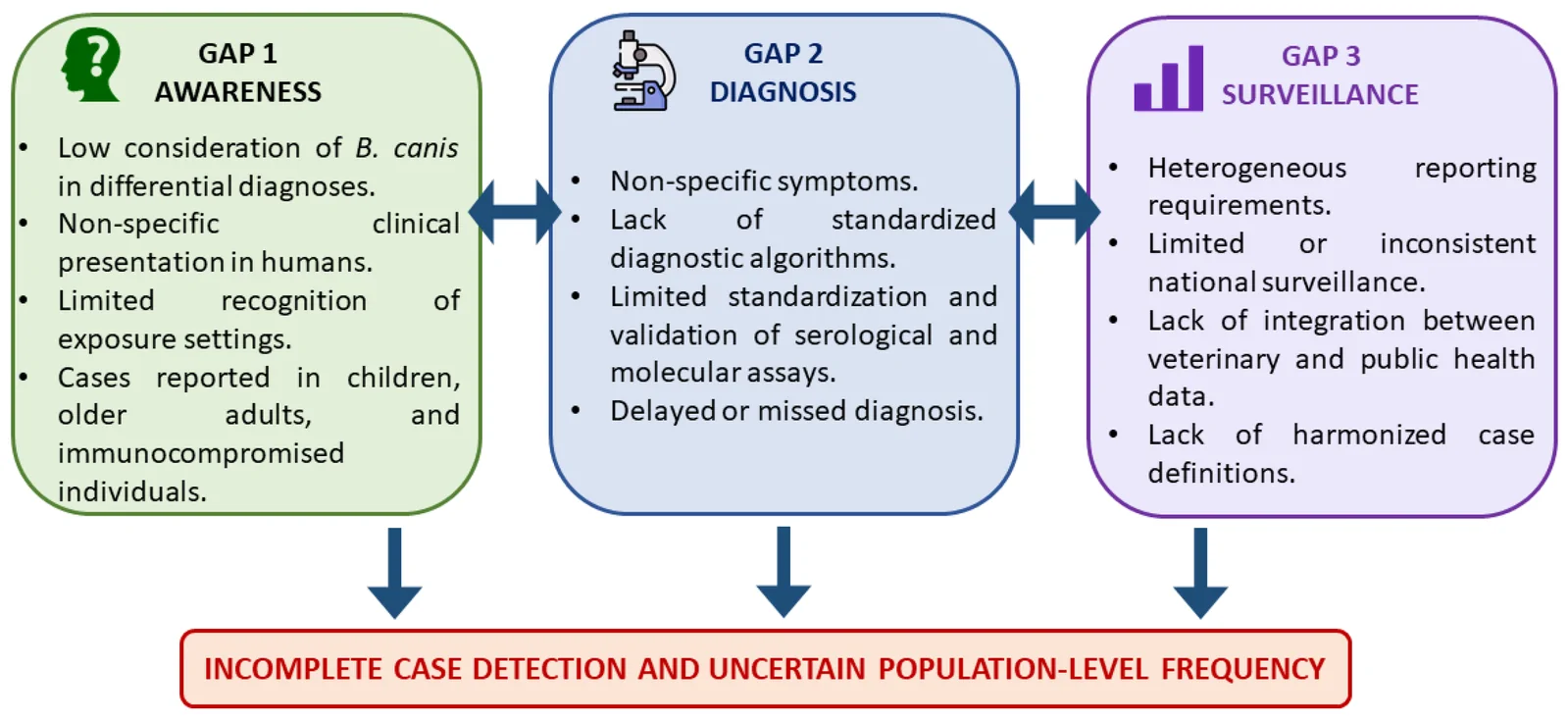
Major gaps limiting recognition of human *Brucella canis* infection. Reduced clinical awareness, diagnostic limitations, and fragmented surveillance may interact to contribute to underrecognition and uncertainty regarding the population-level frequency of human infection.

Recognition of human *B. canis* infection is affected by several interconnected factors rather than a single diagnostic or epidemiological limitation (Figure 4). Low clinical awareness may reduce suspicion among healthcare providers, particularly because infection often presents with nonspecific manifestations and may occur outside recognized occupational exposure settings [1,2,3]. These awareness gaps are compounded by important diagnostic limitations, including the lack of standardized human diagnostic algorithms and limited validation of available serological and molecular assays [3,53,78,79]. In parallel, heterogeneous surveillance and reporting practices make the population-level frequency of human infection difficult to estimate [1,3], while limited integration between veterinary and public health sectors remains an important One Health challenge [26,81]. Together, these three gaps may contribute to delayed diagnosis and incomplete case detection.

## 8. Regulatory and Surveillance Frameworks

Regulatory and surveillance approaches to *Brucella canis* differ substantially between jurisdictions. Within the European Union, Regulation (EU) 2016/429 provides the overarching framework for the prevention and control of transmissible animal diseases, while Directive 2003/99/EC establishes a framework for monitoring zoonoses and zoonotic agents [82,83]. However, the current EU list of diseases subject to specific Animal Health Law rules identifies infection with *B. abortus*, *B. melitensis*, and *B. suis*, but does not specifically list *B. canis* [84]. Consequently, these instruments do not themselves establish a harmonized EU-wide surveillance or control programme specifically for canine or human *B. canis* infection.

National approaches may therefore differ. In Great Britain, detection of *B. canis* in dogs became reportable during 2021 [85]. More recently, Great Britain introduced mandatory pre-import *B. canis* testing for commercially imported dogs originating in or dispatched from Romania, effective from 7 October 2025, illustrating how surveillance and import measures may be adapted in response to changing national risk assessments [86]. These differences highlight the absence of a uniform international approach and the importance of interpreting surveillance data within the regulatory context in which they are generated.

## 9. Conclusions

The available evidence indicates that the principal challenges associated with *Brucella canis* are no longer limited to recognition of its zoonotic potential, but increasingly concern the ability to detect, monitor, and manage infection consistently across veterinary and human health settings. A key priority is the standardization and validation of *B. canis*-specific diagnostic reagents and assays, particularly for serological and molecular testing, together with improved comparability of diagnostic results across laboratories.

Important surveillance gaps also remain. Within the European Union, *B. canis* is not specifically included among the listed diseases subject to harmonized Animal Health Law control measures, limiting the development of coordinated EU-wide surveillance and control strategies [84]. Greater attention should therefore be given to risk-based monitoring of companion animals involved in non-occupational exposure settings, including household dogs and animals moving through rescue, adoption, breeding, and international transport networks. Integration of veterinary screening, human exposure assessment, laboratory capacity, and information exchange within a One Health framework will be essential to improve detection and provide a stronger evidence base for future surveillance and prevention policies [81].

## Figures and Tables

**Table 1:** Summary of published human *Brucella canis* infections and their principal epidemiological and clinical characteristics.

Year (Reference)	Country	Exposure	Patient(s)	Clinical Presentation	Diagnosis	Confirmation (Culture/Serum Only)	Key Feature
1969 [15]	USA	Laboratory exposure	2 laboratory workers	Mild illness to systemic illness with bacteremia	Blood culture; agglutination	Culture-confirmed	Early documented laboratory-associated human infections
1972 [63]	USA	Dog exposure/contact	23-year-old woman	Fever, chills, and pharyngitis	Blood culture	Culture-confirmed	Culture-confirmed human *B. canis* infection
1975 [64]	USA	Non-laboratory exposure: household dog identified as likely source in one case	2 patients	Fever, chills, malaise, and weight loss	Culture/serological methods	Culture-confirmed	Non-laboratory-associated human infection; probable household dog source in one case
1975 [65]	USA	Dog contact	48-year-old man	Intermittent fever and bacteremia	Blood culture	Culture-confirmed	Prolonged bacteremia over 4 months
1978 [66]	USA	Dog contact	Adult woman	Febrile systemic illness with bacteremia	Blood culture; negative *B. canis* agglutinins	Culture-confirmed	Culture-confirmed infection despite negative serology
1999 [67]	USA	No specific animal exposure reported	Adult male	Endocarditis	Serology (presumptive diagnosis)	Serum only	Presumptive *B. canis* endocarditis
2000 [68]	USA	Dog exposure	2 adolescent boys	Aortic valve involvement and infected aneurysms in one patient; calvarial osteomyelitis, epidural abscess, pleural effusions, and pulmonary nodules in the other	Serology/agglutination testing	Serum only	Unusual focal manifestations in adolescents
2004 [16]	Argentina	Laboratory exposure	Laboratory worker	Recurrent fever, headache, arthralgia, weakness	Blood culture; serology/agglutination	Culture-confirmed	Laboratory-acquired infection associated with inadequate biosafety
2005 [53]	Argentina	Positive culture or close contact with culture-positive dogs	17 sera from patients with positive culture or close contact with culture-positive dogs	Brucellosis-like symptoms	Culture; RSAT; iELISA	Mixed evidence: culture-confirmed and serology/exposure-based	Evaluation of human serological diagnosis
2010 [69]	Argentina	Dog exposure	HIV-positive adult	Prolonged febrile illness	Blood culture	Culture-confirmed	Immunocompromised patient
2010 [54]	Argentina	Close daily household contact with infected bitch and puppies	Family cluster involving 6 persons	Mild febrile illness	Culture; serology	Culture + serology	Family cluster including children
2010 [70]	Japan	Occupational exposure to aborted canine placenta	2 pet-shop workers	Fever/fatigue with bacteremia	Blood culture; PCR; serology	Culture-confirmed	Occupational transmission associated with reproductive material
2015 [17]	USA	Pet-store puppy	3- year-old child	Bacteremia	Blood culture	Culture-confirmed	Pediatric case with major laboratory exposure
2018 [13]	Iran	Dog exposure	Elderly woman	Prolonged febrile illness	Blood culture; PCR	Culture-confirmed	Diagnostic delay
2019 [71]	Japan	Dog exposure	39-year-old woman	Chronic intermittent fever/fatigue	Serology (SAT)	Serum only	Long-standing chronic infection in Japan
2021 [18]	Canada	Household dog	Elderly woman	Bacteremia	Blood culture	Culture-confirmed	Secondary laboratory exposure
2022 [52]	Netherlands	Dog breeding facility;/direct contact with placenta and other reproductive	55-year-old woman	Fever, malaise, headache	Blood culture; confirmatory species identification	Culture-confirmed	First Dutch human case: transboundary dog movement implicated epidemiologically
2025 [72]	USA	Exposure to delivery of stillborn puppies	Pregnant 17-year-old	Pyelonephritis-like presentation	Blood culture; species confirmation	Culture-confirmed	Pregnancy-associated human *B. canis* infection following reproductive exposure
2024 [73]	Japan	Dog exposure	68-year-old man	Meningoencephalomyelitis/neurobrucellosis	Serum tube agglutination + abnormal CSF; blood/CSF cultures negative	Serum only; cultures negative	Severe neurological manifestation
2025 [74]	South Africa	Ownership of dogs used for hunting small animals	Adolescent	Febrile adolescent male with bacteremia	Blood culture/isolation, MALDI-TOF genus-level, species-specific PCR, whole-genome sequencing	Culture-confirmed	First laboratory-confirmed human case in South Africa
2020–2024 [75]	Chile	Predominantly current or previous dog exposure	10 patients	Prolonged fever, spondylodiscitis, myopericarditis, meningoencephalitis, hepatitis and lymphadenopathy	ME-RSAT serology; blood cultures negative in tested patients	Serum only; cultures negative in tested patients	Recent multicenter human case series highlighting predominantly dog-associated exposure and serology-based diagnosis

**Table 2:** Advantages and limitations of the principal laboratory methods relevant to the diagnosis of human *Brucella canis* infection.

Method	Main Advantages	Main Limitations	Representative References
Blood culture	Definitive diagnosis; species identification; isolate available for further characterization	Limited sensitivity due to intermittent bacteremia; prolonged incubation; biosafety risk during laboratory manipulation	[16,17,59]
Rapid Slide Agglutination Test (RSAT)	Rapid, inexpensive screening test	Limited specificity; false-positive reactions; requires confirmatory testing	[50,53]
2-Mercaptoethanol RSAT (2-ME RSAT)	Higher specificity than RSAT; useful confirmatory assay	Limited availability; still requires careful interpretation	[50]
Indirect ELISA (iELISA)	Improved sensitivity and specificity compared with agglutination assays	Lack of international standardization and limited clinical validation	[53,79]
Recombinant antigen ELISA	Promising diagnostic performance; reduced cross-reactivity	Experimental; limited clinical validation	[79]
PCR-based assays	Rapid detection; species-specific identification; useful before seroconversion	Lack of standardized commercial assays; variable laboratory protocols; limited validation	[52,59]

## Data Availability

No new data were created or analyzed in this study. Data sharing is not applicable to this article.
